# Successful Combination Immunotherapy for Postoperative Recurrence of Esophageal Cancer Following Prior Immune Checkpoint Inhibitor Treatment: A Case Report

**DOI:** 10.70352/scrj.cr.26-0111

**Published:** 2026-07-29

**Authors:** Ryo Sakada, Tetsuro Kawazoe, Keita Natsugoe, Yuki Shin, Yoshitaka Imoto, Tomoya Harima, Sho Nambara, Yasuo Tsuda, Tomonori Nakanoko, Koji Ando, Eiji Oki, Tomoharu Yoshizumi

**Affiliations:** 1 Department of Surgery and Science, Graduate School of Medical Sciences, Kyushu University, Fukuoka, Fukuoka, Japan; 2 Department of Advanced Medicine and Innovative Technology, Kyushu University Hospital, Fukuoka, Fukuoka, Japan

**Keywords:** esophageal cancer, immune checkpoint inhibitor, immunotherapy

## Abstract

**INTRODUCTION:**

Esophageal squamous cell carcinoma (ESCC) remains an extremely aggressive malignancy with a poor prognosis, particularly among patients with distant metastases. Although systemic chemotherapy is the standard of care for patients with stage IV disease, conversion surgery has emerged as a promising treatment strategy for selected patients who respond favorably to systemic therapy. The introduction of immune checkpoint inhibitors (ICIs) has increased the therapeutic opportunities. However, the optimal management of postoperative recurrence after ICI-based conversion surgery remains unclear. Here, we report a case of postoperative recurrent ESCC after ICI-based conversion surgery in which long-term disease control was achieved through sequential multidisciplinary treatment, including chemoradiotherapy and subsequent dual immune checkpoint blockade.

**CASE PRESENTATION:**

A 68-year-old woman was diagnosed with stage IVB ESCC (cT3N2M1b) with para-aortic lymph node metastasis. The patient received 3 cycles of pembrolizumab combined with cisplatin and 5-fluorouracil (FP), which resulted in marked tumor regression. She underwent robot-assisted thoracoscopic subtotal esophagectomy with para-aortic lymphadenectomy; an R0 resection was achieved. The pathological diagnosis was squamous cell carcinoma with grade 2b tumor regression. Postoperative adjuvant therapy with nivolumab was initiated. Five months after surgery, locoregional recurrence occurred, and the patient was treated with chemoradiotherapy using docetaxel, followed by combination immunotherapy with nivolumab and ipilimumab. Remarkable regression of the recurrent lesions was achieved, and the patient has remained disease-controlled for 22 months postoperatively without further treatment.

**CONCLUSIONS:**

Dual immune checkpoint blockade with nivolumab and ipilimumab may represent a potential therapeutic option for patients with recurrent ESCC following ICI treatment. This case underscores the importance of individualized treatment strategies and the evolving role of immunotherapy in the multidisciplinary management of patients with advanced esophageal cancer.

## Abbreviations

CPS
: combined positive score
ESCC
: esophageal squamous cell carcinoma
FDG
: ^18^F-fluorodeoxyglucose
FP
: cisplatin and 5-fluorouracil
ICI
: immune checkpoint inhibitor
PD-1
: programmed death-1
PD-L1
: programmed death-ligand 1
UICC
: Union for International Cancer Control

## INTRODUCTION

Esophageal cancer remains one of the most aggressive and lethal malignancies worldwide, with a generally poor prognosis, particularly for patients presenting with distant metastases. Systemic chemotherapy has traditionally been the standard care for these patients because curative resection is typically not feasible.^1)^ Conversion surgery has emerged as an important treatment strategy for select cases with potentially resectable distant metastatic lesions. Several reports have suggested that conversion surgery can result in long-term survival for carefully selected patients, thereby offering a potentially curative option beyond conventional chemotherapy.^2–4)^

In recent years, the advent of ICIs has significantly transformed therapeutic strategies for advanced ESCC. ICI-based systemic therapy—particularly in combination with cytotoxic chemotherapy—has been associated with greater tumor shrinkage, higher response rates, and improved overall survival compared with conventional chemotherapy alone.^5)^ The widespread introduction of ICIs has created more opportunities for patients to become candidates for conversion surgery after initial systemic therapy.

Despite these advances, the optimal management strategy for patients who experience recurrence after conversion surgery remains unclear. Treatment decisions are highly individualized and depend on multiple factors, including the affected site, recurrence extent, performance status, and interval since the last systemic therapy. Currently, no consensus or established guidelines exist for selecting systemic therapy in this setting, particularly when patients have previously received ICI-containing regimens.

The CheckMate 648 trial has provided pivotal evidence regarding the role of immunotherapy in advanced ESCC. The study not only demonstrated the efficacy of nivolumab combined with chemotherapy but also highlighted the potential benefit of dual immune checkpoint blockade with nivolumab plus ipilimumab, which achieved durable responses and prolonged survival in a subset of patients.^6)^ However, the efficacy of readministering ICIs or initiating combination immunotherapy after prior ICI exposure remains unclear.

Herein, we report a case of stage IVB ESCC in which long-term disease control was achieved through multidisciplinary treatment, including ICI-based chemotherapy, conversion surgery, chemoradiotherapy, and subsequent dual immune checkpoint blockade after prior ICI exposure.

## CASE PRESENTATION

**Fig. 1:**
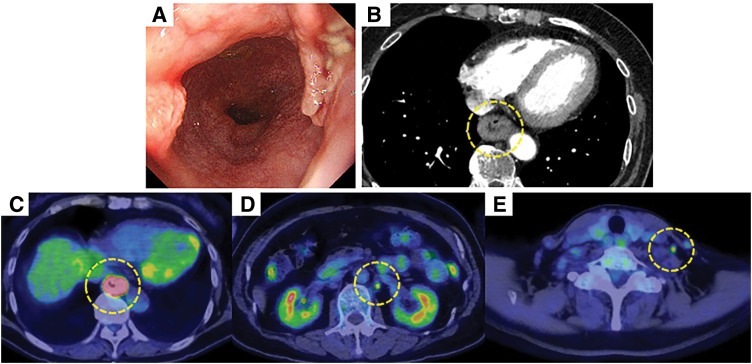
Imaging findings at initial diagnosis. (**A**) Upper gastrointestinal endoscopy reveals a type 2 lesion centered on the posterior wall of the midthoracic esophagus. (**B**) Contrast-enhanced CT demonstrates eccentric, near-circumferential wall thickening of the midthoracic esophagus (yellow circle). (**C**) PET-CT shows increased FDG uptake in the midthoracic esophagus (yellow circle). (**D**) Abnormal FDG accumulation suggesting lymph node metastasis is observed in the left supraclavicular fossa (yellow circle). (**E**) Abnormal FDG uptake suggesting lymph node metastasis is noted in the left para-aortic region (yellow circle). FDG, ^18^F-fluorodeoxyglucose

A 68-year-old woman was referred to our hospital with progressive dysphagia. Upper gastrointestinal endoscopy revealed a type 2 lesion centered on the posterior wall of the midthoracic esophagus (**Fig. 1A**). Biopsy of the lesion was performed, and the pathological diagnosis was well-to-moderately differentiated squamous cell carcinoma. The PD-L1 CPS of the specimen was approximately 6%–8%. Contrast-enhanced CT revealed near-circumferential wall thickening of the midthoracic esophagus (**Fig. 1B**). The left supraclavicular and para-aortic lymph nodes showed FDG accumulation on PET-CT and were considered lymph node metastases (**Fig. 1C**–**1E**). Based on these findings, the patient was clinically diagnosed with stage IVB ESCC (cT3N2M1b, cStage IVB, 9th edition UICC TNM Classification of Malignant Tumours).

**Fig. 2:**
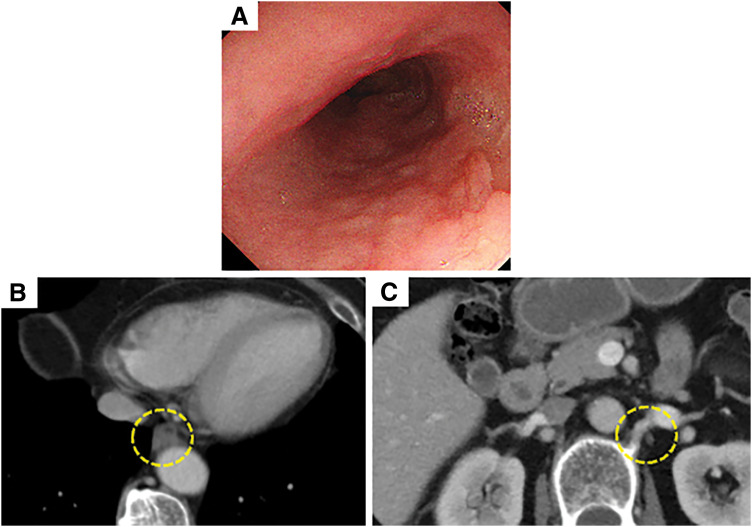
Imaging findings after 3 cycles of pembrolizumab plus FP therapy. (**A**) The tumor in the midthoracic esophagus showing size reduction. (**B**) CT shows improvement in the wall thickening of the midthoracic esophagus (yellow circle). The longest diameter of the primary esophageal lesion decreased from 21 to 12 mm, corresponding to a 42.9% reduction. The overall treatment response was classified as a partial response according to RECIST criteria. (**C**) The para-aortic lymph nodes are decreased in size (yellow circle). FP, cisplatin and 5-fluorouracil

Chemotherapy with pembrolizumab 200 mg combined with cisplatin 80 mg/m^2^ and 5-fluorouracil 800 mg/m^2^ was administered for 3 cycles, each lasting 21 days. Prior to the third cycle, grade 3 neutropenia developed, resulting in a 1-week treatment delay. After 3 cycles of chemotherapy, follow-up CT showed marked reductions in the primary tumor and metastatic lymph nodes (**Fig. 2**). The longest diameter of the primary esophageal lesion decreased from 21 to 12 mm, corresponding to a 42.9% reduction. In the absence of new lesions or unequivocal progression of nontarget lesions, the overall treatment response was classified as a partial response according to RECIST criteria. Given the favorable response and resectability of the metastatic lymph nodes, conversion surgery was planned.

The patient subsequently underwent robot-assisted thoracoscopic subtotal esophagectomy with retrosternal gastric conduit reconstruction, para-aortic lymphadenectomy, and left cervical lymph node dissection, including station 104L, 1 month after the final chemotherapy treatment.

**Fig. 3:**
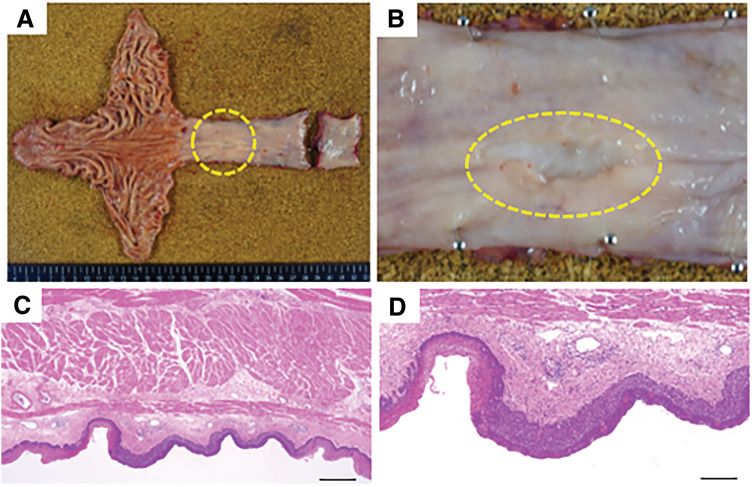
Histological findings. (**A**) Macroscopically, an ulcerative scar measuring 3 × 5 mm at the site of the primary tumor is observed (yellow circle). (**B**) Enlarged view of the lesion (yellow circle). (**C**, **D**) Histopathological examination reveals squamous cell carcinoma with grade 2b tumor regression. (**C**) Original magnification: ×40; scale bar: 500 μm; (**D**) original magnification: ×100; scale bar: 200 μm.

The postoperative course was uneventful, and the patient was discharged 13 days postoperatively. The pathological diagnosis was ypT1a(LPM)N2M1b(#16b), ypStage IVB, with grade 2b tumor regression (**Fig. 3**). One station 104L lymph node was harvested, and histological examination revealed no evidence of metastasis. Postoperative adjuvant therapy with nivolumab (240 mg) every 2 weeks was initiated.

**Fig. 4:**
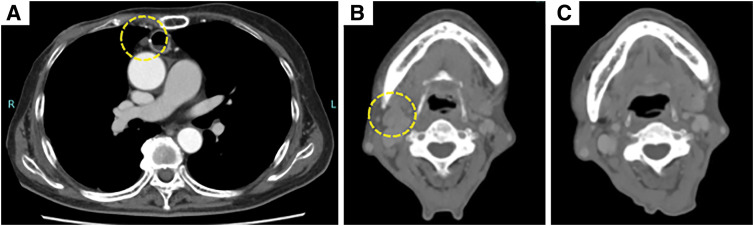
Follow-up imaging after postoperative treatment. (**A**) Five months postoperatively, during nivolumab therapy, metastasis to the right gastric tube lymph node is observed. Radiation therapy with concurrent docetaxel was administered (yellow circle). (**B**) Seven months postoperatively, right cervical lymph node enlargement is noted, and combination immunotherapy with nivolumab and ipilimumab was initiated (yellow circle). (**C**) Ten months postoperatively, a size reduction in the lymph nodes is observed, and the treatment was switched to single-agent nivolumab.

At 5 months postoperatively, recurrence was detected in the lymph node adjacent to the right side of the gastric conduit. No cervical lymph node metastases were evident at that time. As the recurrence was localized, both surgical resection and concurrent chemoradiotherapy were considered as treatment options. However, surgical intervention carried a potential risk of compromising the gastric conduit blood supply. After careful discussion with the patient, concurrent chemoradiotherapy was considered the most appropriate approach. Regarding the selection of the concurrent chemotherapeutic agent, although no significant hematologic adverse events were observed during the preoperative pembrolizumab combined with FP regimen, the patient experienced fatigue, raising concerns about the tolerability of further FP-based chemotherapy. Therefore, weekly docetaxel was selected as the concurrent chemotherapeutic agent. Ultimately, concurrent chemoradiotherapy consisting of weekly docetaxel (10 mg/m^2^) and radiotherapy (50.4 Gy) was administered (**Fig. 4A**). CT performed 7 months postoperatively revealed a newly developed right cervical lymph node metastasis (**Fig. 4B**). Because a new metastatic lesion developed during local treatment, systemic therapy was initiated. Although the patient had previously been treated with an anti-PD-1 antibody, the preoperative pembrolizumab plus FP regimen had achieved a favorable response, and there was no prior exposure to an anti-CTLA-4 antibody. Therefore, combination immunotherapy with ipilimumab and nivolumab was selected. Ipilimumab (1 mg/kg) was administered on days 1 and 22, and nivolumab (360 mg) on day 1 of a 42-day cycle. Ipilimumab was administered for 2 cycles, after which shrinkage of the right cervical lymph node metastasis was observed. Although continuation of dual immune checkpoint blockade was considered, the patient expressed strong concern regarding the toxicity profile of prolonged combination immunotherapy. Therefore, after careful discussion with the patient, ipilimumab was discontinued and nivolumab monotherapy was continued as maintenance therapy.

Follow-up CT at 10 months revealed a significant size reduction in the recurrent lesions, and treatment was continued with nivolumab monotherapy (**Fig. 4C**). Nivolumab was administered for 9 cycles. At 20 months postoperatively, nivolumab was discontinued due to grade 3 immune-related bullous pemphigoid. The patient has been under observation without further treatment, and as of 22 months postoperatively, her disease has remained controlled without evidence of disease progression. This outcome corresponded to 16 months after nivolumab plus ipilimumab therapy initiation and 11 months after conversion to nivolumab monotherapy.

## DISCUSSION

This report describes a rare instance of stage IVB esophageal cancer with distant metastases. The patient underwent ICI-based chemotherapy, achieved significant tumor reduction, and subsequently underwent conversion surgery with R0 resection. Following surgery, local recurrence was treated with radiotherapy and docetaxel, followed by dual immune checkpoint blockade (nivolumab + ipilimumab), which resulted in a clinical response.

Recently, following the introduction of ICIs, conversion surgery has become feasible—even in cases of stage IVB esophageal cancer—and several reports have suggested the potential for long-term survival in selected patients.^2,7)^ In the present case, sufficient tumor shrinkage was achieved with neoadjuvant chemotherapy including pembrolizumab, enabling conversion surgery with pathological confirmation of R0 resection. The patient had oligometastatic disease involving 2 sites: the left supraclavicular and para-aortic lymph nodes. After 3 cycles of pembrolizumab plus FP therapy, upper gastrointestinal endoscopy and contrast-enhanced CT demonstrated shrinkage of both the primary tumor and metastatic lymph nodes, indicating adequate disease control. The para-aortic lymph nodes were also considered technically resectable. Although conversion surgery for stage IVB disease is not yet established as a standard treatment, previous reports have supported conversion surgery for esophageal cancer with synchronous para-aortic lymph node metastasis as a feasible and promising treatment.^8)^ After providing a thorough explanation to the patient and obtaining informed consent, surgical intervention was performed.

The CheckMate 577 trial demonstrated that adjuvant nivolumab significantly prolonged disease-free survival in patients with stage II/III esophageal cancer following neoadjuvant chemoradiotherapy and R0 resection.^9,10)^ In addition, the JCOG2206 trial investigated adjuvant chemotherapy for patients who fail to achieve a pathological complete response after neoadjuvant therapy. Although definitive evidence supporting the use of nivolumab is lacking in the current case, our patient did not achieve a pathological complete response after preoperative therapy; therefore, nivolumab was administered as adjuvant therapy.^11)^ Several reports on other cancer types have shown that rechallenge with a combination of immune checkpoint blockade—specifically, the addition of a CTLA-4 inhibitor (ipilimumab) to PD-1 blockade—can be effective for patients who develop progressive disease during or after ICI therapy. In particular, patients with metastatic melanoma, cholangiocarcinoma, or hepatocellular carcinoma who were refractory to anti–PD-1 monotherapy subsequently responded to a combination of nivolumab and ipilimumab.^12–15)^ Notably, responses have been observed even in patients with a low or undetectable PD-L1 CPS, suggesting that dual immune checkpoint blockade may exert antitumor activity regardless of PD-L1 status^13)^ (**Table 1**). In the present case, disease progression after chemoradiotherapy was characterized by the emergence of new metastatic lesions during local treatment, prompting consideration of systemic therapy. Although a higher PD-L1 CPS has been associated with greater ICI responsiveness, findings from the CheckMate 648 trial indicate that the overall survival benefit does not necessarily increase in a linear manner with higher CPS values. The patient in the current case had a moderate PD-L1 CPS (score 6–8), not in the very low range (CPS <1). The optimal duration of dual immune checkpoint blockade after prior ICI exposure remains unclear, particularly in postoperative recurrent ESCC. In the CheckMate 648 trial, nivolumab plus ipilimumab was administered until disease progression, unacceptable toxicity, withdrawal of consent, or the end of the trial, with a maximum treatment duration of 2 years in the absence of disease progression or unacceptable toxicity. Therefore, treatment according to this regimen may be considered in patients with advanced ESCC. However, dual immune checkpoint blockade is associated with a risk of immune-related adverse events, and the balance between continued antitumor activity and treatment-related toxicity should be carefully considered in each patient.

**Table 1:** Case reports and series demonstrating the efficacy of dual immunotherapy following progression on prior monotherapy

Case	Diagnosis	Number of cases	Monotherapy (prior ICI)	Adverse event/reason for discontinuation	Dual immunotherapy	Response
Glutsch et al., 2019^12)^	Metastatic melanoma	n = 1	Nivolumab	Nephrotic syndrome	Ipilimumab + nivolumab	Partial response
Kaakour et al., 2022^13)^	Cholangiocarcinoma	n = 3	Anti-PD-1 or PD-L1	Progressive disease	Ipilimumab + nivolumab	Durable responses (PR/CR in some)
Alden et al., 2023^14)^	Hepatocellular carcinoma	n = 32	Anti-PD-1 or PD-L1	Progressive disease	Ipilimumab + nivolumab	PR or disease control in 20%–30%
VanderWalde et al., 2023^15)^	Metastatic melanoma	n = 92 (Ipi + Nivo: n = 69)	Anti-PD-1 or PD-L1	Progressive disease	Ipilimumab ± nivolumab	ORR: ~27%
Present case	Esophageal cancer	n = 1	Nivolumab	Progressive disease	Ipilimumab + nivolumab	Partial response

CR, complete response; ICI, immune checkpoint inhibitor; Ipi + Nivo, ipilimumab + nivolumab; ORR, overall response rate; PD-1, programmed death-1; PD-L1, programmed death ligand-1; PR, partial response

The previously reported cases summarized in **Table 1** also suggest that the treatment duration of dual immune checkpoint blockade after prior ICI exposure has not been standardized. In the reported cases of metastatic melanoma, cholangiocarcinoma, and hepatocellular carcinoma, nivolumab plus ipilimumab was used as salvage therapy after progression on prior anti-PD-1 or anti-PD-L1 therapy. However, the duration of combination therapy, the timing of treatment discontinuation, and the use of subsequent maintenance therapy varied among reports. In some studies, ipilimumab-containing therapy was administered for a limited number of induction cycles, followed by anti-PD-1 maintenance therapy, whereas in others, treatment was continued until disease progression or unacceptable toxicity.^12–15)^ These findings indicate that the optimal duration of dual immune checkpoint blockade remains undefined and should be individualized according to tumor response, toxicity risk, patient preference, and overall clinical condition.

In the present case, shrinkage of the right cervical lymph node metastasis was observed after 2 cycles of ipilimumab-containing therapy. Because the patient expressed strong concern regarding the toxicity profile of prolonged combination immunotherapy, ipilimumab was discontinued after careful discussion, and nivolumab monotherapy was continued as maintenance therapy. Although the optimal timing of de-escalation to nivolumab maintenance therapy remains unknown, this individualized approach may be reasonable in selected patients who achieve tumor control and wish to reduce the risk of toxicity. Further accumulation of cases is needed to determine the optimal duration of dual immune checkpoint blockade and the appropriate timing of transition to maintenance therapy in recurrent ESCC after prior ICI exposure.

Another noteworthy aspect of this case was the treatment sequence; radiotherapy combined with docetaxel was administered before dual immune checkpoint blockade initiation. Radiotherapy not only induces direct tumor cell death but also promotes the release of tumor-associated antigens and immunogenic cell death, potentially enhancing the antitumor immune response through the activation of tumor-specific T cells. This may synergize with the immune checkpoint blockade to achieve greater therapeutic efficacy. The so-called “abscopal effect,” in which tumor regression occurs at sites distant from the irradiated field, has been observed in several cancer types in the context of ICI and radiotherapy combination therapy.^16,17)^ Radiotherapy might therefore have enhanced the effectiveness of immunotherapy. Long-term disease control in this patient may have been achieved through the sequential multimodal treatment strategy, including surgery, chemoradiotherapy, postoperative nivolumab, and subsequent dual immune checkpoint blockade. Therefore, the precise therapeutic contribution of nivolumab plus ipilimumab alone cannot be clearly isolated. However, this treatment sequence may represent a potential therapeutic option for selected patients with postoperative recurrent esophageal cancer after prior ICI exposure.

## CONCLUSIONS

This case suggests that long-term disease control may be achieved through individualized multidisciplinary treatment in selected patients with stage IVB ESCC. Although the therapeutic contribution of nivolumab plus ipilimumab alone cannot be clearly determined, dual immune checkpoint blockade after prior ICI exposure may represent a potential therapeutic option for carefully selected patients with recurrent ESCC.

## DECLARATIONS

### Availability of data and materials

The datasets generated and analyzed during the current study are available from the corresponding author upon reasonable request.
